# Supplementary material for: Generalized linear models provide a measure of virulence for specific mutations in SARS-CoV-2 strains
Source: PLoS One. 2021 Jan 26;16(1):e0238665. doi: 10.1371/journal.pone.0238665 (PMC7837476; doi:10.1371/journal.pone.0238665)
Supplement: S1 Fig — (DOCX) [file pone.0238665.s001.docx]

**A**

**B**

**C**


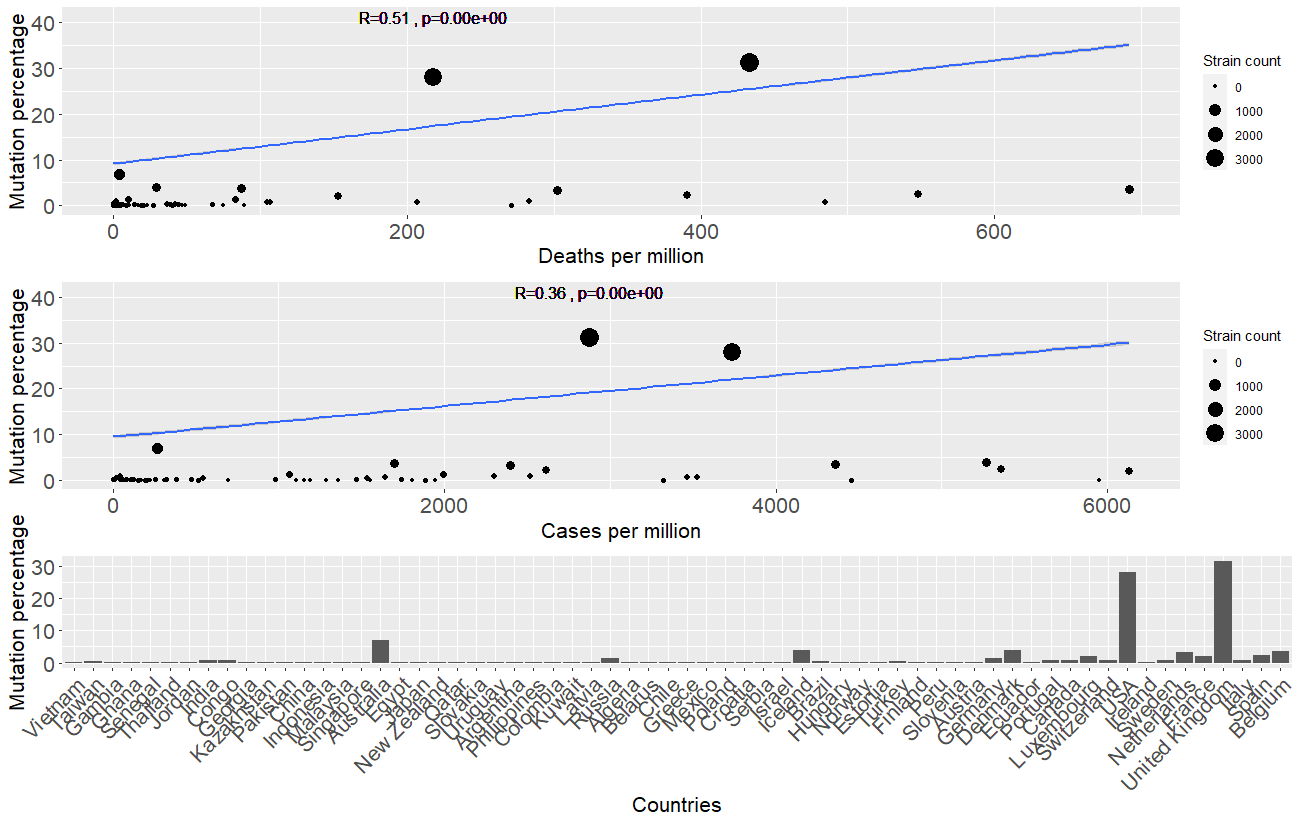


**S1 Fig**. **Analyses plots for S protein mutation at position 23403 (D614G)** **A**. Regression model line showing the simplified fit for mutations percentage across countries and the death rate per million for each country. Pearson’s correlation is shown by the *R* value accompanied by the *p*-value of the correlation coefficient. **B**. Similar regression fit for mutations percentage across countries this time showing cases per million for each country. **C**. Detailed histogram of the percentage occurrence of the mutation across different countries. Countries are sorted with increasing deaths per million.
