# Supplementary material for: Generalized linear models provide a measure of virulence for specific mutations in SARS-CoV-2 strains
Source: PLoS One. 2021 Jan 26;16(1):e0238665. doi: 10.1371/journal.pone.0238665 (PMC7837476; doi:10.1371/journal.pone.0238665)
Supplement: S2 Fig — (DOCX) [file pone.0238665.s002.docx]

**
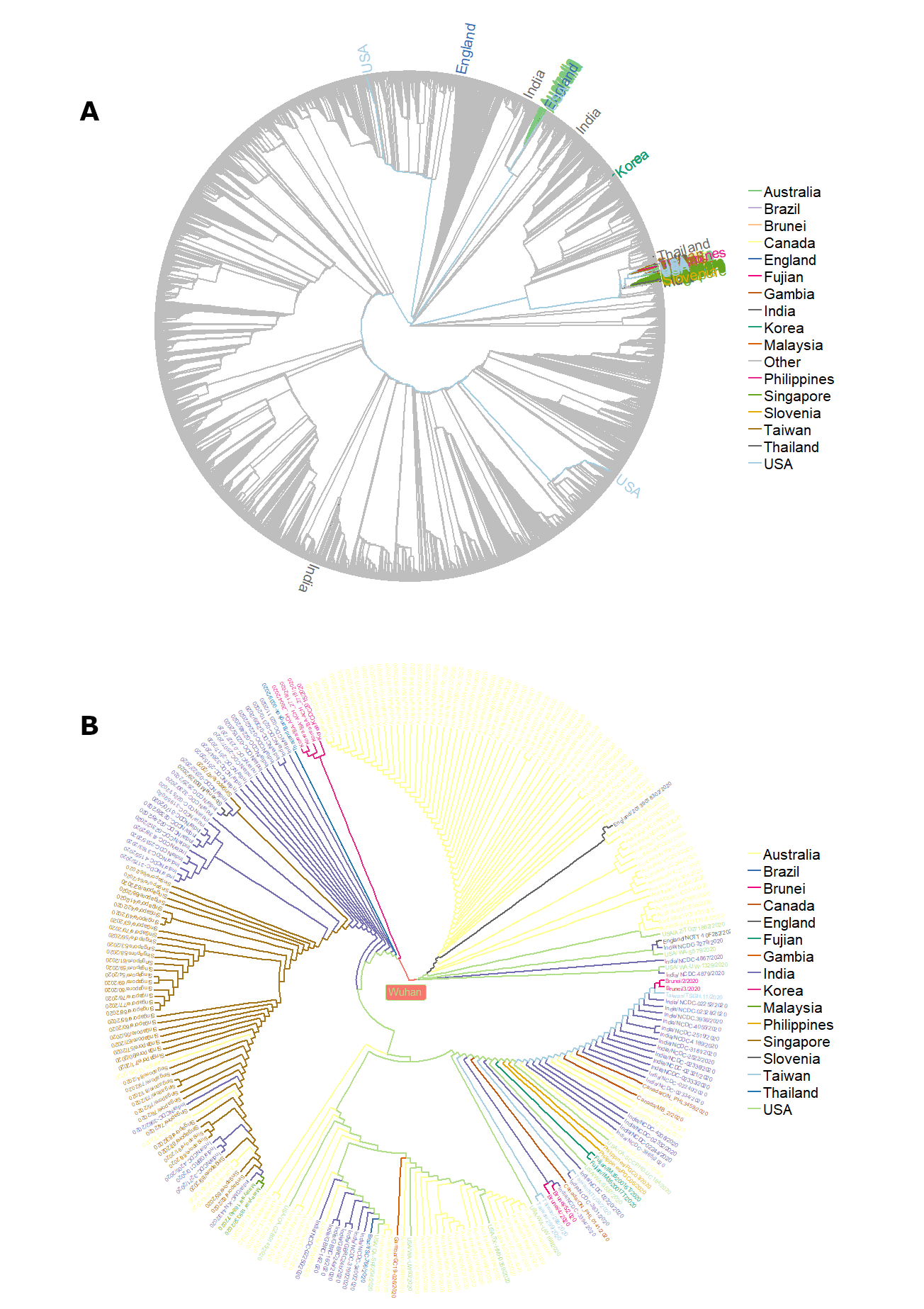
**

**S2 Fig. Phylogenetic Trees using 16,535 trimmed full genome SARS- CoV-2 strains from GISAID**. **A.** A maximum likelihood (RAxML) tree, exapandig form the Wuhan reference strain, with the strains comprising the P13L clade highlighted in colour. Branches of the tree are colour coded by country. **B.** Close up of the P13L clade expanded to show patterns of the P13L mutation that is being tracked across the phylogenetic tree. Branches of the tree are colour coded by country.
