# Supplementary material for: Generalized linear models provide a measure of virulence for specific mutations in SARS-CoV-2 strains
Source: PLoS One. 2021 Jan 26;16(1):e0238665. doi: 10.1371/journal.pone.0238665 (PMC7837476; doi:10.1371/journal.pone.0238665)
Supplement: S3 Fig — (DOCX) [file pone.0238665.s003.docx]

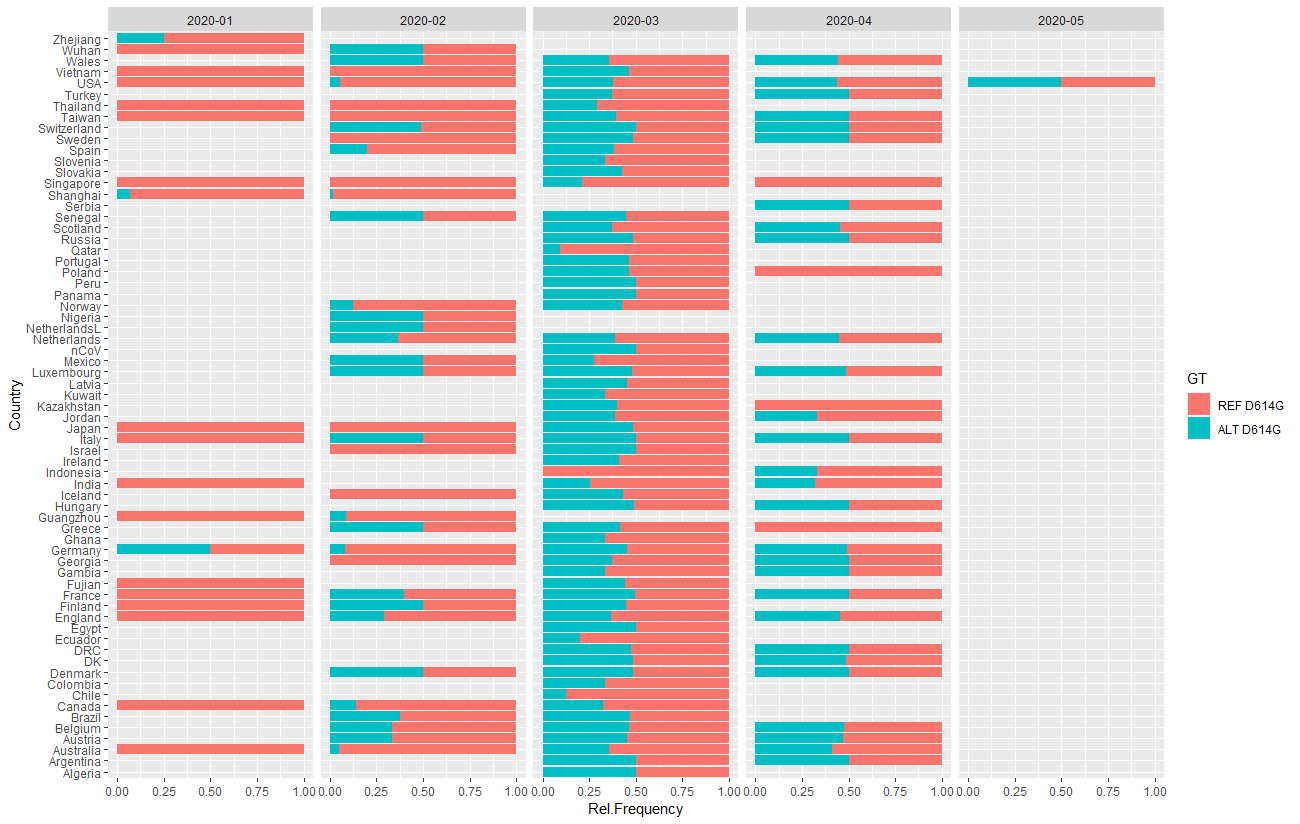


**S3 Fig. D614G transmission in months from its first occurrence in Europe (Germany) to its transmission across the globe**.
