# Supplementary material for: Generalized linear models provide a measure of virulence for specific mutations in SARS-CoV-2 strains
Source: PLoS One. 2021 Jan 26;16(1):e0238665. doi: 10.1371/journal.pone.0238665 (PMC7837476; doi:10.1371/journal.pone.0238665)
Supplement: S4 Fig — (DOCX) [file pone.0238665.s004.docx]

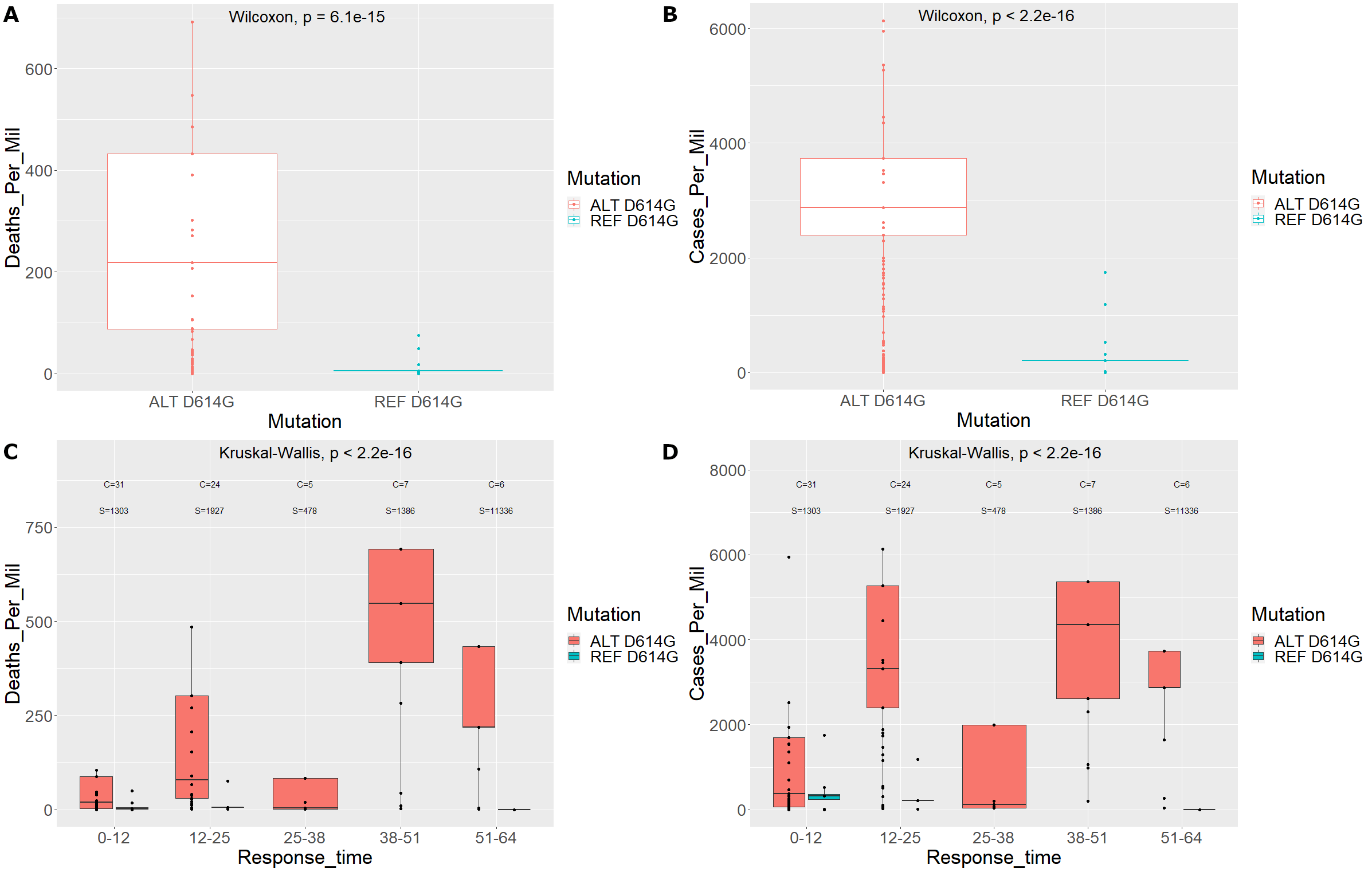


**S4 Fig**. **Boxplot distributions with and without the D614G mutation**. **A.** Deaths per million for countries with the D614G mutation and the reference mutation. **B.** Cases per million for countries with the D614G mutation and the reference mutation. **C.** Deaths per million for countries with the D614G mutation and the reference mutation including response time separation. *C* denotes the number of unique countries in the group and *S* is the number of strains in the group. **D.** Cases per million for countries with the D614G mutation and the reference mutation including response time separation. *C* and *S* are as denoted for panel **C**.
